# Microbiome changes following immigration: Rethinking “Westernization” as an explanatory concept

**DOI:** 10.1080/19490976.2026.2756705

**Published:** 2026-11-10

**Authors:** Riya Sharma, Lola E. Holcomb, Suzanne L. Ishaq, Kieran C. O’Doherty

**Affiliations:** a Department of Psychology, University of Guelph, Ontario, Canada; b Graduate School of Biomedical Science and Engineering, University of Maine, Orono, ME, USA; c School of Food and Agriculture, University of Maine, Orono, ME, USA

**Keywords:** Health, immigration, microbiome changes, Westernization

## Abstract

An emerging focus in microbiome science is on changes to people’s microbiomes following immigration, particularly to so-called Western countries, such as the USA, Canada, and Australia. Moving to these regions has been linked to microbiome changes that may increase immigrants’ susceptibility to health problems, including obesity and Inflammatory Bowel Disease. The term “Westernization” (and adjacent terminology) is frequently used to describe changes in diet, lifestyle, and built environment – and the consequent microbiome shifts – associated with immigration. However, what Westernization means in this context, and whether it is an appropriate descriptor, is often unclear. The purpose of this article is to provide a review of the diverse meanings associated with the terms “West”, “Western”, “Westernization” (and similar) across microbiome-related research and broader uses. We argue that these terms can obscure the specific structural, environmental, and cultural factors shaping microbiome changes. We discuss the consequences of this imprecision and provide recommendations for future research. Avoiding oversimplified conclusions about what impacts immigrants’ microbiome and health can contribute to more accurate targets for public health interventions for these groups in the long term.

## Introduction

A longstanding aim of public health research is understanding how inequalities in social and environmental conditions impact the health outcomes of different groups. In line with this goal, one important strand of research is focused on uncovering the so-called *biological embodiment* of inequitable social conditions (e.g.^1–3^). This relates to identifying biological pathways and mechanisms through which socially stratified experiences can become embedded in human physiology and contribute to health disparities. One such plausible biological pathway that has gained increasing recognition and research attention over the past two decades is the human microbiome, which is now widely considered our “second genome” (see^4–6^). A microbiome refers to a characteristic microbial community occupying a particular habitat, together with its interactions and the environments in which they live.^7^ In humans, distinct microbiomes are found in sites such as the gut, oral cavity, skin, and urogenital tract.

As research on the human microbiome has advanced, so too has our understanding of how people’s microbiome responds to shifts in diet, environment, and lifestyle, and the impact of these shifts on their health.^8–15^ The factors impacting the microbiome change significantly when individuals migrate; people undergo changes in their environments, daily routines, diet and eating rituals, access to support and healthcare, and several other aspects of living.^16^ As such, an emerging area of research focuses on the changes to people’s microbiomes that occur with migration, particularly when people move to highly industrialized countries such as the USA, Canada, and Australia.

Immigration to industrialized nations has been associated with worsening health outcomes among most immigrant groups, which poses a significant public health challenge. A substantial body of epidemiological research on immigrant populations has shown that a longer duration of residence in destinatin countries is associated with increased susceptibility to a range of adverse health outcomes, including asthma and allergies,^17^ obesity,^18^ ^,^ ^19^ and inflammatory bowel diseases.^20^ While microbiome composition can vary substantially both between individuals and within individuals over time,^21^ studies of immigrant populations have also identified some stable changes associated with long-term settlement in the destination country and across generations, suggesting that these patterns cannot be understood solely as short-term temporal variability.^22^ ^,^ ^23^

Microbiome scientists have recently begun to examine changes, particularly in the gut and oropharyngeal microbiomes, as potential biological mechanisms driving the negative health outcomes during and post-migration. In this body of research, the term “Westernization” is frequently used to describe changes in diet, lifestyle, and built environment – and the consequent changes to microbiomes – associated with migrating to a new country. Additionally, the terms “Western” or “Westernized countries” are used to describe the USA, Canada, New Zealand, Australia, and some parts of Europe, and comparisons are made between long-time residents and immigrants to these regions (for instance^23^) or indigenous persons of these regions (for instance^24^).^1^

While there is growing evidence about the potentially harmful consequences of these microbiome changes upon migration to these countries, what exactly is meant by Westernization in this context, and whether it is an appropriate or precise descriptor, needs to be interrogated. To make a case for this undertaking, we provide a brief conceptual overview of the terms “West,” “Western,” and “Westernization” to demonstrate that they encompass a very broad range of meanings.

## Meanings of West, Western, and Westernization

Our aim in this section is to demonstrate that the term “West” and its related linguistic variants (such as Western nations, Westerners, Western populations, Westernization) are not unchanging, natural categories/processes; rather, they rely on social categories whose meanings are fluid and context-sensitive. We do not provide an exhaustive historical review of the concepts; rather, the overview is meant to illustrate why microbiome researchers should avoid these terms in favor of more precise descriptions that reflect specific, observable processes affecting immigrant populations in different countries.

Ifversen^27^ identifies three distinct meanings associated with the term West. When people employ the term, they could be referring to a specific political category, a cultural entity, or a process of Westernization that is presumed to be related to any of these supposed categories. As a political category, the “Political West” referred primarily to the United States and Europe as a transatlantic alliance against the Soviet Union. Yet this meaning was historically contingent: by the 1990s and especially following the Iraq War, scholars questioned whether the United States and Europe still constituted a unified political West (see^28^ ^,^ ^29^).

Beyond geopolitics, “West” and “Western” are also used to signify a particular kind of cultural entity (sometimes referred to as “Western Civilization”). This way of thinking about West extends beyond North America and Europe to include Australia and New Zealand, incorporating nations that are assumed to share certain core ideas and values. Examples of the presumed core ideas and values are Greco-Roman traditions, Christianity, Enlightenment ideals, capitalism, democracy, modernity, and feminism.^27^ However, in this sense, Japan has been described as increasingly westernizing in terms of food pathways, evolving feminist thought, modernity, and capitalism following World War 2^30^ Westernization. As such, these criteria do not map neatly onto geography. Bonnett^31^ argues that “the West is a highly expandable category” (p. 5), often including regions with vastly different historical and cultural backgrounds (e.g., Hawaiʻi, Nunavut, Malta, and Mexico). Moreover, many so-called Western nations now have ethnically diverse and religiously pluralistic populations, making simplistic classifications increasingly untenable.^32^ Similarly, the term "non-Western" faces the same conceptual problems, as it does not refer to a single, homogeneous group. Even within a single country in Asia, for example, there can be stark differences depending on whether study participants are selected from urban or rural areas, forested regions that could have very different vegetation and varying degrees of industrial market access (see, for instance, the sample in the empirical anthropology research by^33^). The categories of Westerner and non-Westerner cannot account for these internal variations. The third meaning associated with West or Western is in reference to the process of “Westernization”. The term is often used to refer to the spread and dominance of the cultural, economic, and political models associated with the so-called West, which were described above.^27^ This concept is associated with multiple social processes, such as the spread of individualistic values, capitalist reforms, shifts in gender roles and family dynamics, among others.^34^ ^,^ ^35^ Using the term to describe biological changes – such as shifts in the microbiome – risks conflating these biological processes with broader societal processes. Additionally, microbiome studies often use the term Westernization interchangeably with urbanization or industrialization, even though these latter processes are not exclusive to America or Europe. Moreover, these processes are not tied to each other in a straightforward way. For instance, Gollin et al.^36^ discuss that while historical patterns in Europe show that urbanization was closely tied to industrialization (i.e., factories drove city growth), many developing countries (e.g., Kuwait, Saudi Arabia, Libya, Nigeria, etc.) became highly urbanized despite lacking significant industrial development. Describing such different processes collectively as “Westernization” therefore obscures which specific changes are actually being observed.

In summary, in contrast to common usage, terms such as West, Western, and Westernization do not denote stable categories or processes; rather, they are fluid concepts with a myriad of meanings. Therefore, their use in microbiome research risks importing dynamic and context-sensitive social meanings into scientific explanations where they become vague and inconsistent. Below, we review how these descriptions occur in microbiome research and highlight the consequences of this imprecision.

## Review process and analysis

This paper is not intended to be a systematic review of all studies that use Western-related terminology in microbiome research. This is because our aim was to critically examine how terms such as “Western,” “Westernized,” and “Westernization” are typically used and what conceptual problems they present. While we attempted to identify and review as many relevant studies as possible, this paper is not based on an exhaustive or representative sample of the literature.

We identified studies through a search of Google Scholar by combining terms such as “microbiome,” “microbiota,” “Western,” “Westernized/Westernised,” and “Westernization/Westernisation.” We also checked the reference lists of relevant papers and used Connected Papers to find related studies. We only included studies on immigrants’ microbiome changes, i.e., research on people who moved from a population described as non-Western to a so-called Western country. We did not include studies that discussed “Westernization” within a single country or population, such as studies describing changes in diet or urbanization as making a microbiome more Westernized within a particular country (e.g.^37^ a study on urbanization in China correlating with a more Westernized microbiome). We also excluded studies that used “Westernization” primarily to describe dietary or lifestyle transitions in different populations but did not investigate associated changes in the microbiome (e.g.^38^). Overall, approximately 15 studies were identified that met our inclusion criteria, although the use of these terms is much more widespread across the microbiome and broader health literature.

Two problems are apparent in the use of these terms within the reviewed literature. Firstly, the terminology is vague in that “Westernization” is used to refer to many different processes lumped together, which do not always co-occur, into a single, catch-all label. These include changes in immigrants’ diets, lifestyle, and environment, which can include varied specific changes such as medication use, exposure to pollutants in air and water, and animal contact, among many others.^39^ ^,^ ^40^

Secondly, the socio-cultural and structural conditions that shape microbiome changes post-immigration are often obscured using broad terms such as “Western diet” or “Western lifestyle.” These labels imply a uniform shift in microbial exposure following migration, and do not reflect the vast differences in social, economic, and environmental conditions within and across countries typically labeled as Western. Microbiome-relevant factors such as diet, housing, access to healthcare, green space, or antibiotic exposure are not just matters of individual behavior, but are deeply influenced by structural determinants such as income, geography, and institutional access.^41^ For example, financial and geographic barriers can limit access to nutritious food or biodiverse environments, reducing exposure to beneficial microbes and increasing health risks.^42^ ^,^ ^43^ Immigrants may experience additional challenges, including cultural adaptation stress, economic disadvantage, and restricted healthcare access, all of which can shape their diet, stress physiology, and living conditions and, in turn, their microbiomes.^4^ These challenges also differ widely between and within different host countries. By attributing microbiome shifts to “Westernization,” research risks masking these important structural dynamics and flattening diverse lived experiences into an overly simplistic framework.

There is also a third issue that has been identified by scholars previously about the use of these terms. This issue pertains to the ethical implications of contrasting populations by using categories such as “Western,” “non-Western,” and “traditional.” For instance, Benezra^44^ has described race as a “ghost variable” in microbiome research, showing how categories such as geography, nationality, lifestyle, and Western/non-Western can reproduce racialized distinctions even when race is not explicitly mentioned. Scaglioni^45^ recently demonstrated how widespread such categories remain in microbiome research. In a review of 180 studies involving non-Western populations, more than three-quarters relied on comparisons such as “Western/non-Western,” “rural/urban,” or “traditional/modernized.” These findings lend empirical support to Núñez Casal’s^46^ argument that race is embedded in the “experimental system” of microbiome science. She argues that such comparisons are not neutral descriptive categories but can reproduce racialized and colonial forms of classification and position certain populations as biologically distinct or as proxies for an ancestral human past. Ultimately, these and many other scholars (e.g.^47^ ^,^ ^48^) have argued that such categorizations treat the so-called non-Western and Indigenous populations as sources of purported and desired ancestral microbial diversity, which can be researched and used to benefit science and personalized-medicine initiatives within the Western world. A full review of the use of race proxies and related ethical issues is beyond the scope of this paper; nevertheless, this remains an important consideration in the use of West-adjacent terminology in the microbiome literature.

## Section I: How are “Westernization” and similar terms used in microbiome research?

There are several ways in which terms such as Westernized and Westernization appear in studies of human microbiomes. The terms have been used since the early 2000s in microbiome research, where researchers wanted to describe changes in the diet and lifestyle of people beyond so-called Western countries. For instance, a few review studies starting in 2007 discuss the “Westernization of diet” or lifestyle for people living in Japan or Africa (e.g.^49^ ^,^ ^50^), noting that diets had more red meat and saturated fat content than before, and lifestyles were becoming increasingly sedentary. Notably, the authors of these studies discussed such diet and lifestyle changes for people who had not migrated to so-called Western countries. Rather, the process of Westernization was discussed as happening within countries such as Africa, India, and China, and these changes were attributed to factors like the “reach of highly profitable global companies”^49^ p. 1177.

While migration has been discussed independent of microbiome research as contributing to worsening health conditions in immigrants to the U.S., a focus on migration gained momentum in research on the gut microbiome around the 2010s, particularly in relation to the rising incidence and concerns about inflammatory bowel disease (IBD) among immigrants (see^51^). In this section, we present an overview of how these terms are used in immigration-related microbiome research, and for what purpose.

Westernization is often used to describe a shift in the (gut, oral, and other) microbiome of immigrants when they move to Canada, USA, Australia, and specific European countries, such as the United Kingdom and Ireland. We present examples from such research on migration, microbiome changes, and health impacts below. The table included in the Appendix (see Appendix A) illustrates a few examples of language and terminology used in studies to describe the relationship between migration and microbiome changes. The term Westernization and its adjacent terms are used in mainly three ways:

1. To categorize populations based mainly on geography (e.g., “Western countries”, “Westernized populations”).
2. To describe what is happening to the immigrants’ microbiomes (e.g., “westernized gut”).
3. To describe potential causes of microbiome change (e.g., “Western diet”).

In the following sections, we use “immigrant” when referring specifically to individuals who have settled in destination countries that are often labeled Western. We retain the terminology used by the original authors to describe particular immigrant groups. For example, where studies distinguish between first-generation and second-generation immigrants, or use population-specific terms such as “Euro-immigrants”,^52^ we preserve those labels.

### To categorize populations and societies

In microbiome research, terms like “Western populations” are frequently used to characterize groups of people based on their geographic location (living in or moving to specific countries deemed to be “Western”). These terms are often employed to contrast populations of people migrating to North America, Australia, and some parts of Europe with people born in those countries (including second-generation immigrants in those countries). Common uses of the term Western in this way include:

(a) *Use of “Western” to differentiate between place of origin*

The term Western is used to discuss different populations based on place of origin, specifically distinguishing between U.S.-born and foreign-born individuals. In one study, “racially/ethnically diverse” U.S.-born people (Whites, Blacks, and Hispanics) are identified as a Western group, and their gut microbiota were compared to those of foreign-born, immigrant Koreans and Hispanics, who are also referred to as “non-Western immigrants to Westernized countries”^53^ p. 1647. Notably, this Western/non-Western distinction appears only in the discussion section; elsewhere, the study refers to more specific community labels. Additionally, in the discussion section, there is an implied connection between place of origin and microbial composition, linking Western location to specific types of microbial profiles. For instance, higher *Prevotella copri* abundance in foreign-born Koreans and Hispanics is contrasted with lower levels in U.S.-born Whites, implying origin-based strain diversity. Further, the study noted a higher abundance of *Bacteroides plebeius* in foreign-born Koreans (attributed to seaweed consumption) and *Bifidobacterium* in groups prone to lactose intolerance (foreign-born Koreans and Hispanics, U.S.-born Blacks). So, the term Western is linked in that study to presumed origin-based dietary differences.

Similar distinctions based on place of origin and exposure to *Western practices* are made in a study that differentiates among Indians residing in India, Indian immigrants in Canada, Canadians of Indian ancestry (Indo-Canadians), Canadians of European ancestry (Euro-Canadians), and immigrants to Canada with European ancestry from another Western country (Euro-immigrants). In this case, Western practices are defined as “reduced physical activity, changes in hygiene/medical practices, and acculturation to a westernized diet, commonly characterized by caloric dense, highly processed foods with low fiber intake”^52^ p. 2. The implicit assumption here is that the 5 groups differ in their level of exposure to such practices: Indians residing in India having the least exposure, Indo-Canadians and Euro-Canadians having experienced the highest exposure, and the difference in presumed exposure leads to distinct microbiome characteristics. Euro-immigrants were used as a control group, wherein the implicit assumption is that they serve as a representative model of immigrant individuals who have had prolonged exposure to said Western practices.

(b) *Interchangeable use of the term Western to refer to industrialized, high-income, and developed nations, or modernized societies*

The term Western is often used interchangeably with industrialized, high-income, and developed nations, or modernized societies for characterizing nations and societies. For example, in one study comparing Australia and China, the term “Western country” is used to describe the former as a “developed” country characterized by higher levels of hygiene, while China is framed as an “Eastern-developing” country.^54^ The study invokes the *hygiene hypothesis* to explain that lower levels of exposure to pathogens in so-called Western countries such as Australia could contribute to a changed oropharyngeal microbiome, which could be associated with the observed rise in allergy and asthma rates among long-term Chinese immigrants.

Another study on gut microbiome changes in Hispanics living in the United States uses the terms Western and non-Western to refer to a higher-income country (the U.S.) and low- to middle-income countries (Latin American nations), respectively.^55^ It is unclear if Hispanic residents born in the USA (2nd generation immigrants) and those who relocated in childhood and adolescence (1.5th generation immigrants, as per the study) are classified as non-Western, like those who migrated in adulthood (1st generation immigrants).

Regarding such categorization, it would be interesting if the Western and non-Western labels may change based on changes in the microbial profile. That is, will people having a high bacterial and fungal diversity and high Prevotella-to-Bacteroides ratios still be considered non-Western? In fact, there are studies that refer to second-generation immigrants in North America as part of a Westernized population, based on shared microbial features with other U.S. or Canadian cohorts, while people residing in their country of origin or recent migrants are described as Non-Westernized (e.g.^22^ ^,^ ^56^).

### To describe an outcome in the form of a changed microbial profile (“Westernized gut” or “Westernized oropharyngeal microbiome”)

Another common application of concepts related to *Westernization* in microbiome literature is to describe an outcome, namely, a changed microbial profile after migration. Studies that use the term Westernization in this manner typically label gut microbiome profiles as “Western microbiome” and “non-Western microbiome”.^23^ ^,^ ^57^ The transition from the former to the latter is characterized as:

1. Loss of a native strain (i.e., the shift from *Prevotella* to *Bacteroides* dominance).
2. Loss of function associated with the shift in microbial profile (e.g., the loss of enzymes associated with dietary fiber degradation).

The microbiome post-transition is then denoted using the terminology “Westernized microbiome”^52^, or it is stated that immigrants’ gut “westernizes”.^23^ In these studies, this terminology is employed to denote that the microbial composition of immigrants’ gut or oropharynges has shifted to resemble that of the European American/Canadian people born in the United States/Canada. Copeland et al.^22^ similarly used the term “westernized metagenome” (p. 10) to describe a state of microbial composition that is (1) tied to lifestyle and dietary changes following migration for 1^st^ generation Indian immigrants to Canada, and (2) more aligned with that of long-term residents in Canada and America. Through these comparisons, the study frames “westernized” not just as a geographical label but also as a distinct microbiome outcome shaped by exposure to a certain (so-called Western) diet and lifestyle. The characteristics of the diet and lifestyle associated typically with the *Western* label are the focus of *Section I.3*.

Importantly, this terminology overlaps with other descriptors, such as *industrialized microbiomes* or *VANISH.* D’Aloisio et al.^58^ use the acronym *VANISH* (Volatile or Associated Negatively with Industrialized Societies of Humans) to describe a microbiome presumed to be common in people who lived close-to-nature lifestyles, but that has mostly vanished in people living in cities today. Alternatively, BloSSUM (Bloom or Selected in Societies of Urbanization/Modernization) has been described as a microbial profile which is presumed to be more common in urban/industrial/modern environments. All in all, the term Westernized is used as a shorthand for a changed microbiome profile post-migration, such that imprecisely defined social and geographical antecedents are implied to have precise biological consequences.

### To describe broad causal factors of microbiome change (“Western diet”, “Western lifestyle”, “Western environment”)

The third type of application of the terms in microbiome studies is to describe the putative causal factors of microbiome changes, mainly “Western diet” and “Western lifestyle.” Several studies discuss the changes in immigrants’ (gut, oral) microbiomes when they migrate to the United States^23^ ^,^ ^52^ ^,^ ^53^ and Canada^22^ as influenced by their diet. Increasingly, studies suggest that the longer immigrants reside in the U.S. or Canada, the more their gut microbiome shifts to resemble that of individuals born in these countries and long-term residents of these countries.

The gut microbiome’s sensitivity to both short- (week-long;^59^) and long-term (lifestyle >1 y^60^) dietary changes is well established, and this has led many microbiome researchers to identify what they refer to as a Western diet as a key factor in explaining microbial shifts among immigrants. In these studies, a Western diet is *typically* described as a diet that is composed of ultra-processed foods, which are high in sugars, fats, and animal proteins, with low fiber content, which is a consistent use of the term across these studies.^61^ Studies often link this diet to a reduction in gut microbial diversity, an increase in pathogenic bacteria, and associated health issues such as obesity,^23^ diabetes,^55^ and inflammatory diseases.^54^

In contrast, immigrant diets are commonly referred to as *traditional* or *non-Western*. For instance, in a study by O’Keefe et al.^62^ which involved a two-week diet switch between African Americans and rural South Africans, the authors used the terms “Westernization of diet” and “Africanization of diet” to describe the switch. Here particularly, the term Westernization refers narrowly to dietary changes and excludes other potential factors that may cause changes to microbiomes.

Studies that try to link dietary shifts to microbiome changes do so in different ways. Vangay et al.^23^ and D’Aloisio et al.^52^ correlated microbiome data to diet data, and identified specific shifts in diet which were likely responsible for changes in the microbiome, including increased protein, salt, and sugar with decreased fiber,^23^ and specifically a change in the type of oils/fats consumed.^52^ Copeland et al.^22^ and van der Vossen et al.^57^ only used predictions of diet shifts gleaned from differentially abundant microbial biochemical pathways in the data, but they also speculate that shifts in diet are responsible for shifts in the microbiome. Wang et al.^63^ noted that ‘dietary acculturation’ was associated with changes in microbial communities in Hispanics and Latinos who had emigrated to the US, and that the microbial change induced metabolic disease.^63^ Importantly, while all of these studies examined a “Western Diet”, none of them included the exact same diet or observed the exact same results, which tells us that specific diet and microbial shifts are the mechanism by which microbiomes change, and specific terminology is needed to study it.

A few microbiome studies on immigrants also mention the concept of *dietary acculturation.* ^23^ ^,^ ^52^ ^,^ ^53^ While the term is never explicitly defined, its usage across the studies consistently shows that researchers use the term to describe the adoption of a Western diet by immigrants. As we explore in *Section II.2,* this way of conceptualizing dietary acculturation is reductive and imprecise.

Beyond diet, researchers also discuss that the adoption of a “Western lifestyle”, (sometimes simply referred to as “lifestyle”) can contribute to the “Westernized microbiome” of immigrants. While rarely defined explicitly, lifestyle is most often characterized in terms of:

a) Physical activity levels (and presumed reduction as people immigrate to countries of interest)
b) Changes in hygiene practices and medical practices, such as use of antibiotic and antiparasitic medications.^52^

Thus, in this third application, the term Westernization signifies a presumed process whereby immigrants’ diets and physical activity levels (“lifestyle” factor) shift to resemble those of Euro-Americans or Canadians (depending on the study), though the precise nature of activity of the latter groups is often not examined.

It is important to note that there is no study which reports on the gut microbiome of people who migrate but are able to maintain their typical diet; thus, we cannot fully disentangle migration from dietary shifts. However, studies of short-term travel could be used as a proxy, as geography and diet change during travel, but other changes to microbial exposure, which are inherent to immigration, would not be major drivers of the microbiome. For example, changes in the daily microbiome of a researcher are easily traced to travel-related changes in diet and health, reverting back once the travel was completed.^60^ Similarly, all travelers to Peru were found to have changes to their gut bacteria over their stay of 10–100 d, regardless of whether they experienced “traveller’s diarrhoea” or not, and regardless of their diet.^64^ Diarrhoea was associated with the depletion of 4 species of commensal gut bacteria and the increase in *Escherichia coli*, a known pathobiont, but was not associated with diet.^64^ To study the effect of short-term changes in diet related to travel, a trial used feces from healthy people in 6 geographic areas to “humanize” the gut microbiome of gnotobiotic (germ-free) mice, then changed their diet to simulate travel to another geographic area.^65^ Not only did diet shifts change the gut microbiome, but diet changed gut motility and transit through the gastrointestinal tract, which was posited to be responsible for some of the changes in the microbiome through changes to biochemistry and physics of digestion, which open/close nutrient niches.^65^ Collectively, these studies point to a loss of commensals in the gut, which allows for either non-native commensals or pathobiont/opportunistic species to establish in the gut. The studies discuss this loss of commensals as responsible for changes in health related to travel and migration, which are mediated by changes in the diet.

In summary, we showed in this section that Westernization and related terms are being used in microbiome studies on migration to denote geographical, biological, and socio-cultural shifts. Further, the term Westernization is used synonymously with processes implicated in microbiome alteration, such as industrialization, modernization, urbanization, and (dietary) acculturation. The problems with drawing on these concepts and terms in such a manner are discussed in the next section.

## Problems with the use of Western-related terminology

When terms like Westernization are used in the ways which are described in Section 1, at least two problems follow: (1) there is a loss of precision when studies collapse various microbiome changes into broad category or process labels, and (2) such usage obscures the broader environmental and behavioral dimensions beyond dietary change that may contribute to microbial shifts in immigrant populations.

### Loss of precision

Many studies begin with a specific focus, such as the disappearance of a particular gut microbial strain following migration or changes in microbiome diversity. However, as findings are interpreted and discussed, the term Westernization is often introduced as a shorthand for describing observed microbiome shifts. Sometimes the term is placed in quotation marks, perhaps to acknowledge that it functions more as an informal or catch-all descriptor than a precise scientific category.

As discussed in Section 1, across the literature, this pattern appears in work focusing on diverse immigrant populations. For instance, some studies report increases in the Bacteroides to Prevotella ratio, and the associated loss of carbohydrate-active enzymes like *β*-glucanases, or declines in the gut microbial diversity of Southeast Asian immigrants to the U.S.^23^ Similarly, shifts in the abundance of taxa such as *Dialister succinatiphilus and Prevotella copri* have been linked to changes in fiber intake and increased consumption of processed foods in Indian immigrant cohorts in Canada.^52^ In an oral microbiome study with Chinese immigrants to Australia, researchers note an increase in certain taxa (e.g., *Leptotrichia*) and a decrease in others (e.g., *Deinococcus*) for long-term immigrants.^54^ Although the findings of these different studies vary significantly in their details and contexts, they all use the label Westernization as a kind of shorthand (often after carefully outlining specific microbial changes), perhaps to offer a familiar or accessible frame.

Labeling such diverse changes as Westernization risks conflating different health outcomes and population-specific factors. These outcomes range from deteriorating immune responses in Chinese immigrants to Australia^54^ to patterns observed in Latin American immigrants in the U.S.^55^, where microbiome changes were more closely linked to obesity.

Additionally, findings suggest that microbiome shifts in immigrants vary by immigrant group in terms of health implications. Peters et al.^53^ assessed US immigrants’ gut microbiomes across racial/ethnic groups and found significant differences among Korean and Hispanic immigrants, with each group losing distinct microbiota as they adopted more “Westernized dietary” patterns. For example, Koreans showed a marked decrease in *Bacteroides plebeius*, a bacterium associated with seaweed digestion. This change did not align with Hispanic immigrants; it was associated with Korean dietary shifts specifically. As such, the catch-all term Westernization risks diluting meaningful differences between health outcomes for different groups.

Terms like “Western environment” suffer from similar problems. Guo et al.^54^, for instance, use it “as a collective term to denote the socio-cultural, physical and geographical environment in developed countries, such as the United States and Australia, and the ‘Eastern environment’ as a collective term to denote developing countries, such as China” (p. 6). It is difficult to categorize the dietary practices of China and other so-called developing countries as neatly Western or Eastern. Adding broad-level qualifiers such as east or West environment, developed or developing, or industrialized or non-industrialized obscures the substantial diversity within and between countries.

Importantly, differences in how categorization is determined can alter which designation a country receives, resulting in further loss of precision when conducting meta-analysis. The conversation surrounding whether China and its dietary customs are “Westernized” or “Non-Westernized” is a good example. While there have been significant shifts in dietary patterns over the past few decades, primarily a transition towards the so-called Western dietary habits as characterized by increased consumption of animal products and processed foods.^66^ This dietary transition signifies not only changing food preferences but also broader sociocultural and economic transformations that influence nutritional health and food systems in China. A critical aspect of this transition is the marked increase in the consumption of animal-based foods, including meat and dairy, which contrasts sharply with China’s historical dietary patterns that predominantly featured plant-based foods. Research has documented that per capita consumption of animal products has risen significantly, moving away from a traditional diet that was low in meat to one increasingly characterized by a Westernized dietary pattern high in red meat and other animal-based foods.^67^ ^,^ ^68^ As such, it would be important to rethink the categorization of Chinese immigrants’ diet as “non-Western” and contrast it with the host country “Western diet.” A similar consideration is required for studies on Korean immigrants, since typical diets vary amongst different Koreans. Shin et al.^69^ have compared three kinds of dietary patterns common among Koreans living in Korea: a typical Korean diet, a typical American diet, and the NHANES 2001–2004 recommended-healthy American diet. Their study also found a change in the gut bacterial communities which correlated to changes in nutrient intake and connections to health status, though there was no geographical shift to the U.S.

### Missing aspects of microbiome “Westernization” beyond specific diet changes

As described in *Section I.3,* when the term Westernization is used in microbiome studies, it is most often linked to immigrants’ dietary changes or acculturation: consuming a diet higher in processed food, refined sugars, and fats, often characterized as a “Western diet”. There are two problems in discussing dietary changes as “non-Western” to “Western” food and labeling that as Westernization.

Firstly, the so-called dietary acculturation of immigrants after the shift is much more complex than eating or not eating certain foods. This is evident in, for instance, Vangay et al.’s study, wherein even after Hmong immigrants moved to the US, their rice consumption was similar to that of second-generation Hmong, and these groups consumed 10 times more rice than the US-born European Americans in the study. Similarly, Nadkarni’s^70^ study on first-generation, ethnically diverse immigrants to Canada shows that they continue to prefer and eat their ethnic food. In fact, immigrants’ *dietary acculturation* leads to them combining multicultural foods in Canada with the food from their home countries, i.e., an integrated diet, which cannot be labeled Western or non-Western in a simplistic way.

Another dimension of dietary acculturation is that it goes beyond just *what* people eat; it also includes changes in *how* people eat, such as the timing and frequency of meals. For example, East Asian immigrants in the US and Canada displayed altered breakfast and meal schedules after migrating (see ^71^), which could potentially lead to changes in the gut microbiome, though the evidence currently remains tentative.^72^ Another factor related to *how* people eat is the food preparation method, which has been shown to impact the gut microbiome. Hauptmann et al.^73^ show that traditional drying methods in Greenlandic Inuit culture led to distinct microbiomes in dried fish, which differed from the dried fish prepared industrially. Their findings emphasize how traditional food preparation and local environmental conditions shape unique microbial profiles, suggesting that these influences might be lost or homogenized when industrial methods replace traditional ones. Changes in eating schedules and changes in food preparation cannot all be captured in the overly broad concept of Westernization. Food preparation changes could be more accurately described as outcomes of industrialization, though even that does not cover all changes with great precision. For example, the industrialization of food systems can involve changes in food processing, packaging, and agricultural production, including greater exposure to food additives (emulsifiers, artificial sweeteners) and toxicants (pesticides and fungicides) which may also influence the gut microbiome.^74^

Importantly, food systems vary both across countries and within geographic regions, including differences in food availability, processing, distribution, and consumption patterns.^75^ Accordingly, these features should not be assumed to characterize all “Western” settings uniformly. Baker et al.^76^ have also highlighted that the spread of ultra-processed foods and mass food production methods increasingly affects countries globally. Conflating industrialization (of food production) with “Westernization” could falsely imply that these changes are exclusive to the so-called West.

Secondly, a focus on dietary acculturation alone neglects other potential changes associated with migration to and life in the host countries.^2^ Examples include changes in exposure to immigration and post-immigration stress, municipal drinking water, antibiotic use, and treatments with antiparasitics, all of which could also lead to microbiome changes. These factors are typically acknowledged by the authors as important to consider, though this acknowledgment is typically found in the concluding sections of studies (see^5^ ^,^ ^77^) without integrating these possible interpretations into analyses (contra^23^). Therefore, the impact of these factors on microbiomes has not yet been systematically investigated with respect to immigration. That said, there is one ongoing study by Kim et al. (study protocol^77^) that aims to study both biological and psychosocial mechanisms associated with depression and anxiety among Chinese and Korean immigrants in the United States. The conceptual framework (p. 3) used by the authors suggests that gut microbiomes may be shaped by physiological stress or inflammation caused by psychosocial factors such as chronic stress, discrimination, and acculturative stress faced by immigrants.

Like dietary environments, the other factors described above vary considerably within the so-called Western host countries (i.e., within and across America, Canada, Australia). For example, the experience of post-immigration stress can differ drastically depending on the socio-economic context, the immigrant's region of origin, the specific host country within which they reside, and even the specific location within the host country.^79^ ^,^ ^80^ Another consideration to be noted is that there are different healthcare systems and practices across these nations.^81^ This diversity across the so-called West makes it difficult to apply a singular term like Westernization to account for all the diverse factors that shape microbiomes in immigrant populations. Similarly, dietary patterns among immigrant populations are influenced not only by the broader national context but also by local food availability in different regions within a country. For instance, immigrants in less diverse areas may face challenges in accessing fresh fruits and vegetables, and specific ingredients, leading to different patterns of dietary acculturation​.^82^ ^,^ ^83^ While the loss of home-related foods is somewhat universal, the adoption of new foods and whether fresh food or junk food is selected is not.^83^

Furthermore, one’s diet also depends on socio-economic conditions (see^66^ ^,^ ^84^) and the food environment itself,^85^ which can differ dramatically within a country. Food insecurity is also responsible for negative health outcomes in migrant populations to the US,^83^ who often face additional logistical barriers in their attempts to access fresh and healthy foods.^82^

Additionally, migration experiences may also vary by country of origin and characteristics of the migration process itself. Earlier studies indicate that microbiome composition may differ with respect to birthplace, age at time of migration, length of time in the host country, and country of resettlement.^5^ ^,^ ^23^ ^,^ ^55^

However, studies on immigrants’ microbiome changes use *Westernization* to discuss mainly dietary acculturation, and even that is narrowly used to refer to food products consumed and the ultra-processed, packaged nature of the food.

## Discussion and recommendations

As outlined in our introduction, the term Westernization is a fluid and polysemous concept; meanings associated with the term range from political alliances to cultural values and industrial development. Applying it as a catch-all descriptor risks importing this conceptual vagueness into microbiome studies. Similarly, terms like West and Western are not natural or fixed categories; they encompass a wide range of cultural, political, and economic contexts and include regions and populations with significant internal diversity. When these broad and variable terms are used to describe specific microbiome shifts, they risk oversimplifying the distinct environmental exposures, dietary patterns, and social conditions that shape microbial communities in different immigrant groups in different countries. It is important to note that many of the studies we reflect on, as well as others, find statistical significance within their study population when using categorical comparisons of Western versus not. We agree that these comparisons are meaningful in *highly specific* comparisons, because the study populations of people were homogeneously grouped by the number of years or generations in their new country, or had maintained similar diets to each other because of close social interactions within the migrant communities studied. But the categorical terms we use for studying people are never driving the mechanism of change in the microbiome; they simply embody the principles behind the change. Vangay et al.^23^ and D’Aloisio et al.^52^ both found significant changes in the microbiome related to categorical comparisons of Western versus not, but they did not find the same underlying trend in the diet shift. If Vangay et al. had replaced the term “Western” with “low-fibre diets”, their results would still be true, but less applicable to D’Aloisio et al., which found a shift in the oil/fat profile to be important. In fact, when the authors (ANON) randomly assigned Westernization status to human fecal microbiome datasets, they still got significant differences, implying that some other factor was responsible for the differences (see Appendix B). Thus, we again point to the need for specificity in data analysis, rather than the use of broad categories even when those categories are situationally significant.

Research on immigrants' microbiome changes will remain crucial to understanding the health issues linked to migration; therefore, we provide some recommendations for future research, based on the points discussed in this paper:

1. Rather than relying on overly broad labels such as “Western” and “non-Western” diets, it is more useful to examine the specific diet ingredients lost during migration, which remove the nutrient niches for gut commensals. Examining these specific, lost diet ingredients can allow for more targeted dietary interventions for migrant populations to Canada, U.S., or other industrialized countries, which will unlikely be dismantling their industrialized food systems anytime soon. Immigrants to these countries, often face several structural barriers to food security when they move^82^. Thus, there are important implications for public health around food security interventions that can focus on preventing the loss of traditional food sources and the commensals they recruit. Preserving the stability or functionality of microbiomes could preclude the slide towards metabolic disease, something which is already the focus of diet interventions in non-migrant populations, but clearly requires different diet recommendations for migrant populations.
2. It is better to use metabolite/metabolomic data or diet history data, whenever possible, to study the effects of diet on the microbiome in humans. It will also be important not to assume that all diets in a geographic area are homogenous. When these data are not available, using metagenomic data from the gut to identify recently consumed plant and animal DNA^86^ will be useful.
3. Glean metadata from primary sources when using previously published data, particularly in public databases, and do not use broad demographic categories which have been assigned across all studies (e.g., Western) if there is no explanation of how these variables were assessed. More specific geographic data can be integrated from other databases to provide more precise geographic information that would affect microbial exposures (e.g., air pollution, greenspace).^87–89^
4. Consider the implications of diet/lifestyle change and migration within the context of the population studied, and their specific microbial communities, instead of generalizing the trends in microbiome shifts, which may not be relevant for all migrant populations or all regions/diets within a country. Integrating social science and bioethnography research into microbiome studies can provide these contexts through in-depth interviews with study participants to reveal granular lifestyle habits which affect microbial exposures (e.g.^90^ ^,^ ^91^).

Finally, one possible response to the concerns raised in this paper could be to retain “Westernization” as a broader concept while addressing its limitations through more precise definition and operationalization of the particular processes under study. We have considered this approach. Based on the problems identified in this paper, however, we remain cautious about whether the term can be defined in a way that is sufficiently precise, comparable, and conceptually coherent across studies. Nevertheless, the development and empirical testing of more explicit operational definitions could provide a valuable contribution to this debate.

## Conclusion

Existing microbiome research on migration provides valuable insights into the effects of migration on microbiomes, which has important implications for immigrants’ health. However, the use of overly broad terms such as Westernization and social categories of Western and non-Western populations may obscure the specific biological changes being identified and, consequently, make it more difficult to determine where interventions should be directed. In this paper, some recommendations are offered to move past these catch-all terms. While our paper is based on a non-exhaustive review of microbiome research on immigration, and specifically, immigrants to mostly Canada, the USA, Australia, and Western Europe, the considerations and recommendations we shared can also be applied to broader microbiome and other health research using such terminology.

## Figures and Tables

**Figure B1.**
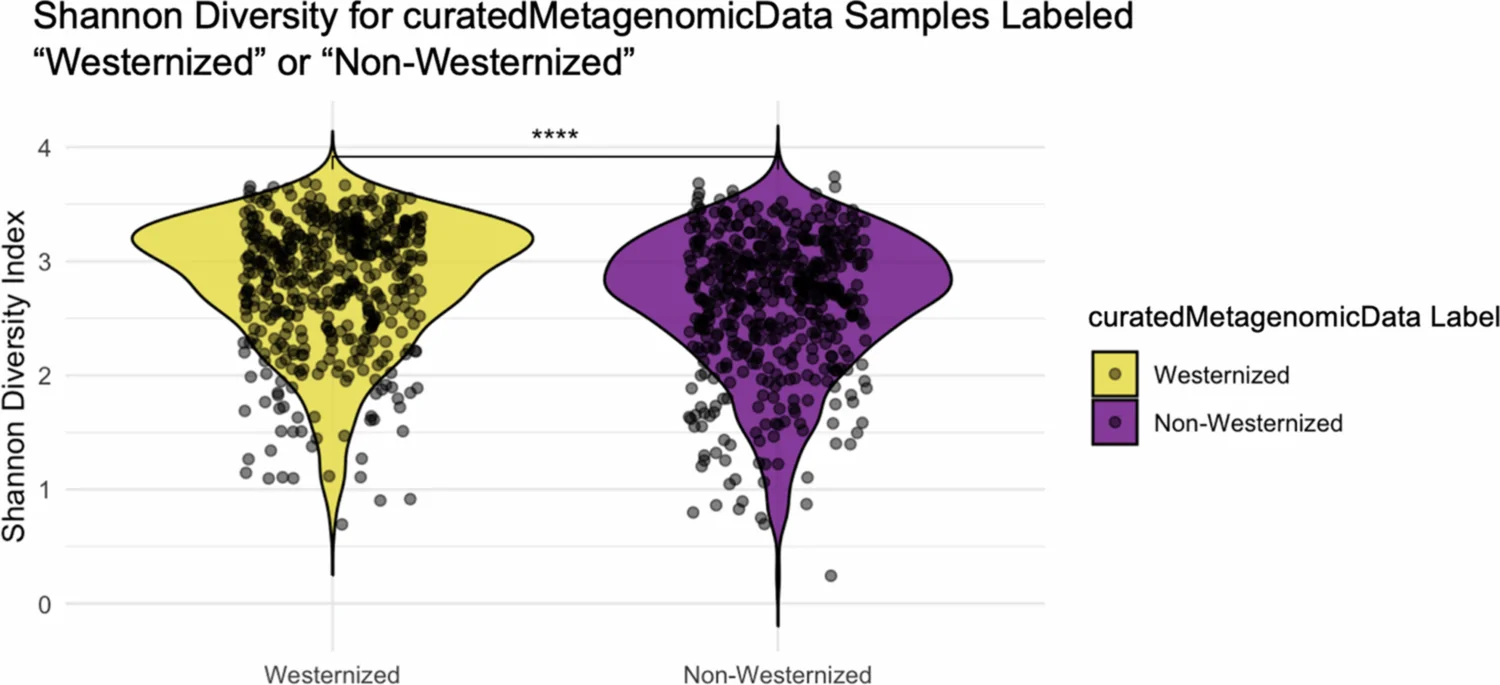
Shannon diversity index for Westernized (yellow) and Non-Westernized (purple) individuals, as designated by the curatedMetagenomicData R package. Graphic made using TreeSummarizedExperiment, mia, and ggplot2 packages in R. Significance added by Wilcoxon rank-sum tests from the ggsignif package in R. Significance code: ****p < 0.0001.

**Figure B2.**
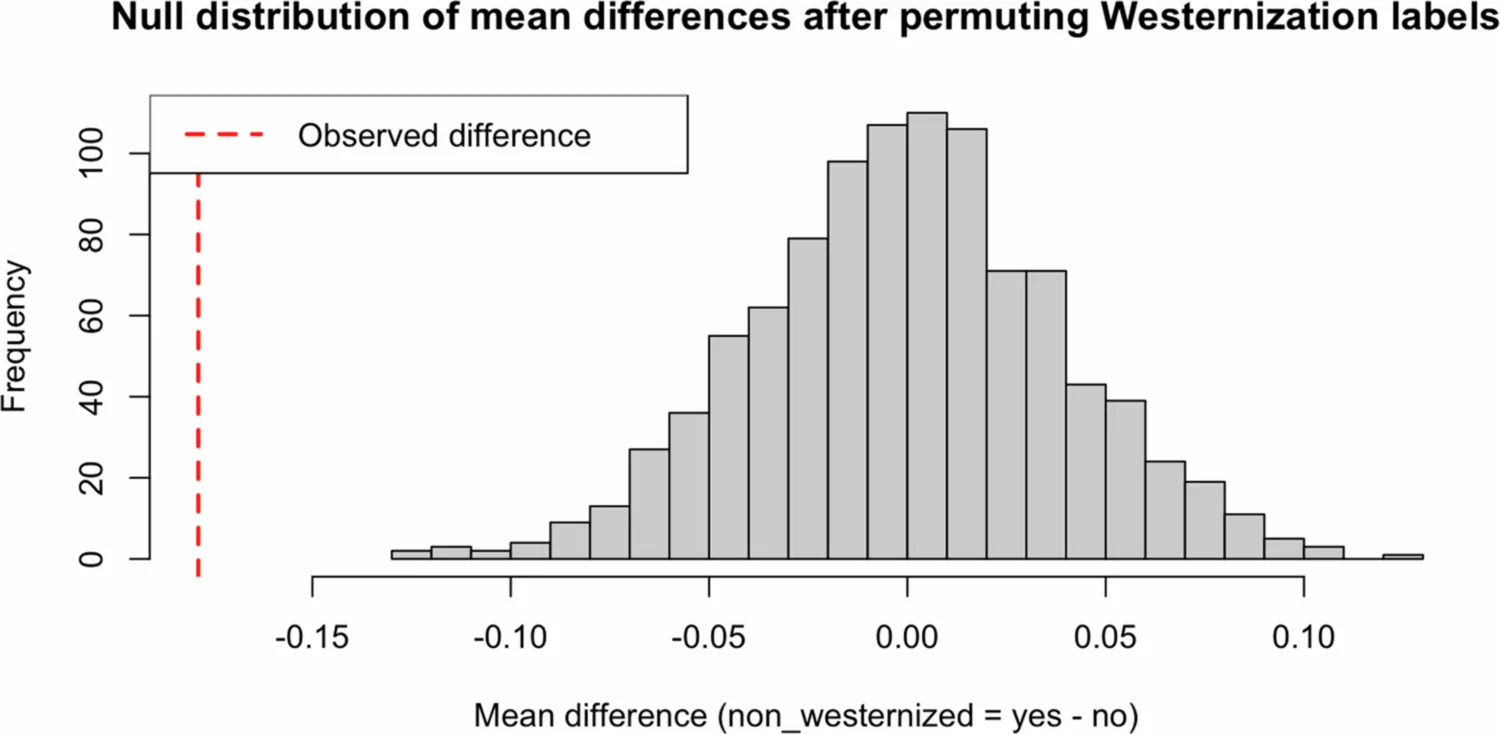
Histogram showing the null distribution of mean differences in Shannon diversity generated from 1000 permutations of randomly assigned Westernization group labels (non_westernized = yes − no). The red dashed line indicates the observed difference from the real data, which falls outside the range of the null distribution (empirical *p* < 0.001), suggesting a statistically significant association between randomly assigned Westernization status and microbial alpha diversity. Formula: mean difference = mean(Shannon, Westernized) - mean(Shannon, Non-Westernized).

**Figure B3.**
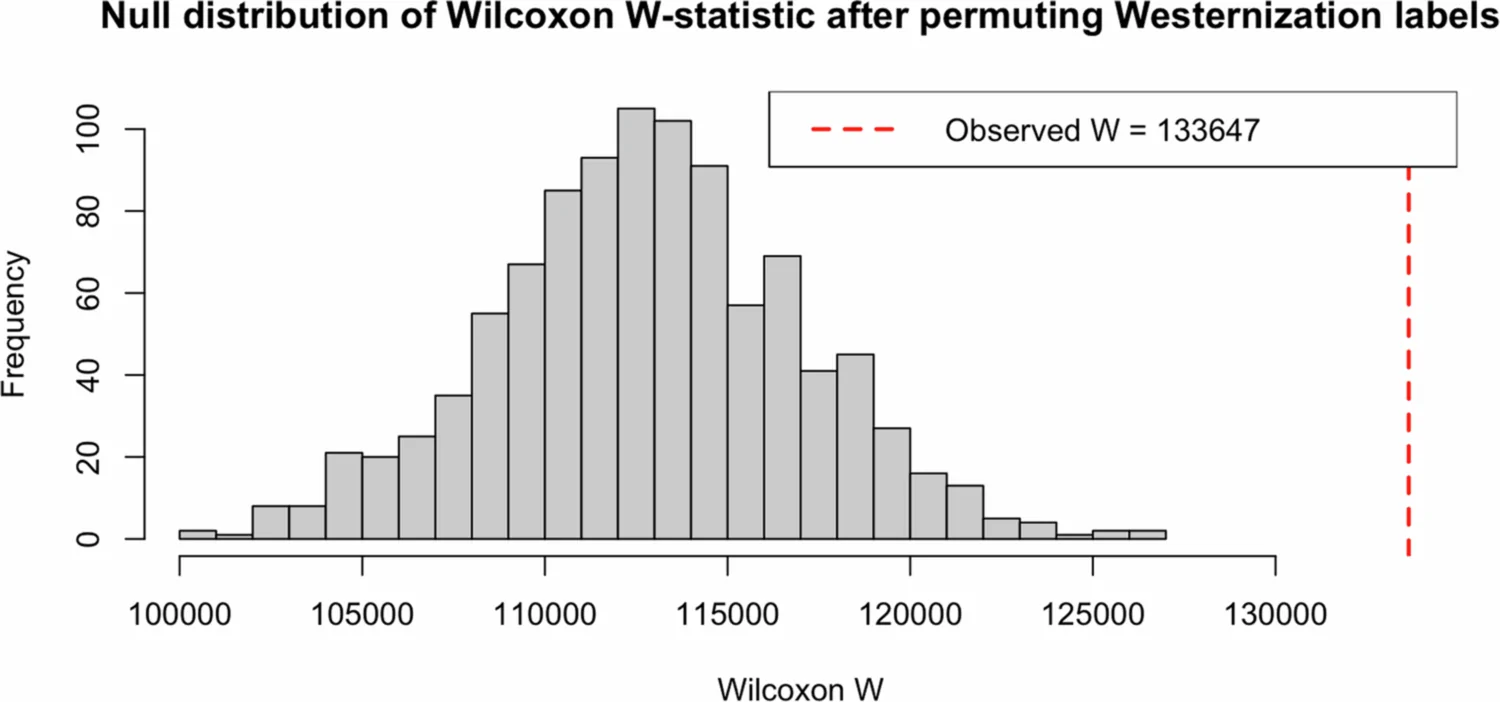
Histogram showing the null distribution of Wilcoxon W-statistics from 1000 permutations of the “non_westernized” group labels. The red dashed line represents the observed W-statistic from the real data. The observed value lies outside the null distribution (empirical *p* < 0.001), indicating a significant difference in Shannon diversity between randomly assigned Westernized and Non-Westernized individuals.

## Data Availability

The data that support the findings of this study are available in ExperimentHub at https://waldronlab.github.io/curatedMetagenomicData/, reference number.^92^ These data were derived from the following resources available in the public domain: https://pmc.ncbi.nlm.nih.gov/articles/instance/5862039/bin/NIHMS941683-supplement-NmethSupplement1.pdf

## Endnotes

^1^
In addition to immigrants, microbiome scientists have shown great interest in comparing microbiomes of the ‘West’ to the so-called ‘traditional’ microbiomes of several indigenous groups of North and South America, Australia, and Europe.^25^ ^,^ ^26^ There is also work on refugee groups. However, the focus of this paper is on research related to immigrant groups to industrialized nations.

^2^
There is a growing body of work using exposomics – the study of cumulative environmental exposures across the life course and their biological effects – to examine how broader environmental conditions may shape the human microbiome (see^78^). A more detailed discussion of this literature is beyond the scope of the present paper.

## Appendices

### Appendix A: Table

**Table:**

Terminology	Usage to denote these processes in specific papers
Westernized (gut, fecal) microbiome.	“The Western-associated genus Bacteroides increasingly displaced the non-Western-associated genus Prevotella across generations in the United States” ([23] *p*. 964)
Western food/dietary regime Dietary acculturation	“the consumption of macronutrients commonly associated with a Western diet: sugars, fats, and protein” ([23] *p*. 966) US nativity and dietary acculturation [53] “Dietary Acculturation Partly Explains Microbiome Acculturation” [23] “The growing immigrant population in North America is rapidly westernizing through the adoption of practices like acculturation of the westernized diet, reduced physical activity and changes in hygiene/medical practices” ([52] *p*. 3)
Western environment	“…use the phrase ‘Western environment’ as a collective term to denote the socio-cultural, physical and geographical environment in developed countries, such as the United States and Australia, and the ‘Eastern environment’ as a collective term to denote developing countries, such as China” ([54] *p*. 6)
Westernized and non-westernized populations	Migration from a non-Western nation to the United States” ([23] *p*. 961) Westernized: European [52] Industrialized Non-westernized: Developing Communities (migrating) from Southeast Asia [22] Traditional populations
Industrialized societies Modern, industrialized populations Societies of urbanization/modernization	“Traditional populations such as the Hadza, a hunter-gatherer group living in Tanzania, like the immigrants from Southeast Asia, harbor microbiota with a larger and more diverse collection of carbohydrate active enzymes (CAZymes) than their industrial counterparts” (​​​​​[56] *p*. 2) “VANISH” (volatile and/or associated negatively with industrialized societies of humans) taxa” ([26] *p*. 2) “The industrialized microbiome has been previously characterized by the decline of VANISH (volatile and/or associated negatively with industrialized societies of humans) taxa, frequently found in populations adhering to traditional lifestyles, and the rise of BloSSUM (bloom or selected in societies of urbanization/modernization) taxa, which appear in modern, industrialized populations” ([52] pp. 1-2)

### Appendix B: Microbiome data and the term “Westernization”

#### Research question

Is the term “Westernization” a meaningful categorical variable for assessing groups of people in microbiome research, or are microbiome differences between samples classified as “Western” versus “non-Western” simply due to random chance? Are the differences biologically meaningful? The basis for this argument is that microbiome analysis often relies on categorization algorithms that are susceptible to differences in sample groups or to variation in effect sizes within groups.

We hypothesized that if “Westernization” status is randomly applied to human microbiome samples, thus creating arbitrary groups of Western/non, these group comparisons will still yield significant *p*-values, indicating that the *act* of creating a category-determined grouping for microbiome samples may artificially create statistical significance, rather than an inherent effect of Westernization on the microbiome. Simply put, if we randomly assign ‘Western’ status and the group comparison yields a significant *p*-value, then the statistical comparison is not really measuring “Western” status at all.

#### Methods

*Data retrieval*

Stool metagenomic datasets and associated metadata from 50 independent studies included in the curatedMetagenomicData R package^92^ were selected based on the following inclusion criteria: healthy (control) study condition, adult (>18 y) age category, and whether samples had been tagged in the database with a Western or non-Western classification (non_westernized = “yes” or “no”). To reduce confounding factors, samples with missing metadata for gender and age or with recorded antibiotic use were excluded. Only baseline or first-collection samples (days_from_first_collection = 0 or NA) were retained to minimize longitudinal effects. Duplicate samples from the same subject were removed. Studies with fewer than 10 samples meeting these criteria were excluded to ensure adequate sample sizes for statistical analysis. Microbial relative abundance data were measured using Shannon-Weaver metric of entropy, which encompasses species richness (abundance) and evenness of the distribution of abundances across taxa. These were retrieved at the species level using the returnSamples() function in curatedMetagenomicData, specifying “relative_abundance” as the data type.

To balance sample sizes across Western and non-Western populations, a stratified random subsampling approach was applied, ensuring equal representation by gender within each population group. The final dataset consisted of 242 samples from each group, for a total of 968 samples in the entire dataset (*n* = 242 Westernized women, *n* = 242 Non-Westernized women, *n* = 242 Westernized men, *n* = 242 Non-Westernized men). The selected sample data span 24 countries and 6 continents.

*Statistical analysis of Shannon diversity by Westernization status*

To assess whether gut microbial alpha diversity differed between individuals previously labeled as Westernized and Non-Westernized, we used Shannon diversity index values and “non_westernized” group labels (yes/no) extracted from the study metadata. All analyses were conducted in R software version 4.4.1.

To determine whether microbial species abundance was statistically significant, we calculated the observed difference in mean Shannon diversity between groups using the existing (“real”) metadata and evaluated its significance (*p* < 0.05) using a linear model. The Shannon diversity index captures both species’ richness and evenness. Higher Shannon values indicate more diverse microbiomes (i.e., more species and/or more balanced in abundance). Shannon diversity is often not normally distributed in host microbiome data, as there are never any samples that lack all microbiota, and there are carrying capacity limits of high diversity. Thus, we conducted a Wilcoxon rank-sum test to compare Shannon diversity, providing a non-parametric assessment of significance.

To test our hypothesis, we performed a permutation test by randomly reassigning “non_westernized” labels across samples 1,000 times while keeping Shannon diversity values fixed. Again, we conducted a Wilcoxon rank-sum test to compare Shannon diversity, providing a non-parametric assessment of significance.

To test whether the difference in microbial species abundance was biologically meaningful, we compared effect sizes to assess the amount of difference between the means in the real and the randomly assigned comparisons. We repeated the Wilcoxon test across 1,000 permutations of randomly assigned group comparisons, recording the W-statistic each time. High W-statistics indicate differences between group means. We used the W-statistics to build a null distribution model in which the randomness of the category of Westernization should create no effect: the W-statistic should be low, and *p*-values should be non-significant. Using the null distribution model compared to the real data, an empirical *p*-value was calculated based on its extremity relative to the null to demonstrate whether the real data W-statistic and the randomly assigned data W-statistic were indicating different effect sizes.

### Results and interpretation

***Using the field-standard statistical approach to assess Shannon Diversity***

We first assessed whether microbial alpha diversity differed between individuals labeled as Westernized or Non-Westernized in the curatedMetagenomicData package, without any re-assignment of the variable. This comparison replicates the typical comparison performed by human microbiome studies, which uses the category of “Western.” Shannon diversity was statistically significantly higher in Westernized individuals compared to Non-Westernized individuals (Figure B1; Wilcoxon rank-sum test, *p* < 0.0001). This is contrary to most studies on the gut microbiome, in which Westernized countries are stated to have lower diversity: we interpret this discrepancy as an example of how data selection plays a role in how Westernization is compared.

The difference in mean Shannon Diversity values between groups was −0.1787638, which, for example, is a 5.58% difference for samples with a Shannon Diversity value of 3.2. To put this in perspective, height is not evenly distributed globally, but height is correlated with higher gut diversity and explains approximately a 1% difference in Simpson’s Diversity.^93^ Similarly, age is not distributed evenly across countries, but age was demonstrated to be a more important factor in determining gut microbial diversity than country of origin.^94^ And recently, bowel movement frequency and composition were statistically significantly correlated with up to 20% lower Shannon Diversity values^95^, and bowel movement frequency is also not evenly distributed across countries.^96^

***Using random re-assignment of “Western” variable to assess Shannon Diversity***

To test whether this association between diversity and Westernization designation could arise by chance, after randomly re-assigning Westernization status to samples 1000 times to compare group means of Shannon diversity. This comparison tests whether any binary grouping of samples will yield a significant *p*-value, or whether only the true “Western versus not” comparison would yield a significant *p*-value. Wilcoxon tests revealed statistically significant *p*-values (<0.05) 59 times.

To test whether this association between diversity and Westernization designation was creating biologically meaningful differences, we performed two permutation-based analyses to compare effect sizes. First, we randomly reassigned Westernization group labels across samples 1,000 times, recalculated the mean difference in Shannon diversity for each iteration, and compared the resulting null distribution to the observed difference (Figure B2).

The distribution of mean differences was centered on 0 (no difference between groups), and the equal tails away from the center indicate that permutational reassignment generated roughly the same amount of comparisons in which Shannon Diversity was higher in the Western group and lower in Western group, however, a mean difference of any size was observed in approximately 800 of the 1000 permutations. The closest effect size generated by the random reassignment was −0.13, which was observed in approximately 5 permutations. For comparison, disease status creates Shannon diversity mean effect sizes up to 0.5 in the human gut,^97^ and location within the intestines of mice an average 1.22 effect size.^98^

Thus, the observed mean difference in Shannon Diversity using the real data (−0.1787638) was more extreme than all permuted mean differences using random reassignment (empirical *p* < 0.001). This finding indicates a statistically significant association between Westernization status and Shannon diversity, even if the effect size was small and could almost be replicated by random chance.

Similarly, in the second permutation test using the Wilcoxon rank-sum statistic (Figure B.3), the null distribution model of W values did not include the observed W-statistic (W = 133,647, empirical *p* < 0.001; Figure B.3). The W-statistics in the null model were evenly centered around W = 113,000, which is 15.45% lower than that of the true model. This again indicates that the effect size is larger in true comparisons of Westernized status, although with a relatively small effect. W-statistic calculations are specific to the data being used and the number of samples, and a direct comparison cannot be made to results from other studies.

### Conclusion

By the numbers, the real value for Westernization provides a statistically significant comparison, although we argue that the effect sizes are so small as to be biologically irrelevant. Together, these analyses demonstrate that the differences in alpha diversity between Westernized and Non-Westernized samples are likely to result from something other than this categorization, as random assignment of this label yielded significant ad hoc group comparisons in many permutations. This suggests that the signal may not be uniquely attributable to the Westernized/Non-Westernized classification itself. Instead, it may reflect broader variability in the human gut microbiome, such as bowel movement frequency,^95^ height,^93^ or age,^94^ where biologically meaningful variables co-vary with Westernization status. Therefore, the observed differences should be interpreted cautiously, as they may arise from underlying microbiome diversity rather than the Westernized/Non-Westernized designation alone. Even the day-to-day changes in abundance and beta diversity within the microbiome of an individual^60^ are larger than the mean differences in Shannon Diversity using Westernization categories.

## References

[1] Bagby SP , Martin D , Chung ST , Rajapakse N . From the outside in: biological mechanisms linking social and environmental exposures to chronic disease and to health disparities. Am J Public Health. 2019;109(S1):S56–S63. doi: 10.2105/AJPH.2018.304864. PMC6356139 30699032

[2] Thorpe RJ, Jr. , Bruce MA , Wilder T , Jones HP , Thomas Tobin C , Norris KC . Health disparities at the intersection of racism, social determinants of health, and downstream biological pathways. Int J Environ Res Public Health. 2025;22(5):703. doi: 10.3390/ijerph22050703. PMC12111065 40427819

[3] Vineis P , Delpierre C , Castagné R , Fiorito G , McCrory C , Kivimaki M , Stringhini S , Carmeli C , Kelly-Irving M . Health inequalities: embodied evidence across biological layers. Soc Sci Med. 2020;246:112781. doi: 10.1016/j.socscimed.2019.112781. 31986347

[4] Amato KR , Arrieta M-C , Azad MB , Bailey MT , Broussard JL , Bruggeling CE , Claud EC , Costello EK , Davenport ER , Dutilh BE , et al. The human gut microbiome and health inequities. Proc Natl Acad Sci U S A. 2021;118(25):e2017947118. doi: 10.1073/pnas.2017947118. PMC8237592 34161260

[5] Fanfan D , Mulligan CJ , Groer M , Mai V , Weaver M , Huffman F , Lyon DE . The intersection of social determinants of health, the microbiome, and health outcomes in immigrants: a scoping review. Am J Biol Anthropol. 2024;183(1):3–19. doi: 10.1002/ajpa.24850. PMC11185843 37737631

[6] Herd P , Palloni A , Rey F , Dowd JB . Social and population health science approaches to understand the human microbiome. Nature Human Behaviour. 2018;2(11):808–815. doi: 10.1038/s41562-018-0452-y. PMC6711373 31457107

[7] Berg G , Rybakova D , Fischer D , Cernava T , Vergès MCC , Charles T , Chen X , Cocolin L , Eversole K , Corral GH , et al. Microbiome definition re-visited: old concepts and new challenges. Microbiome. 2020;8(1):103. doi: 10.1186/s40168-020-00875-0. PMC7329523 32605663

[8] Gilbert JA , Blaser MJ , Caporaso JG , Jansson JK , Lynch SV , Knight R . Current understanding of the human microbiome. Nat Med. 2018;24(4):392–400. doi: 10.1038/nm.4517. PMC7043356 29634682

[9] Ipci K , Altıntoprak N , Muluk NB , Senturk M , Cingi C . The possible mechanisms of the human microbiome in allergic diseases. Eur Arch Otorhinolaryngol. 2017;274(2):617–626. doi: 10.1007/s00405-016-4058-6. 27115907

[10] Kho ZY , Lal SK . The human gut microbiome - A potential controller of wellness and disease. Front Microbiol. 2018;9:1835. doi: 10.3389/fmicb.2018.01835. PMC6102370 30154767

[11] Larsen N , Vogensen FK , van den Berg FWJ , Nielsen DS , Andreasen AS , Pedersen BK , Al-Soud WA , Sørensen SJ , Hansen LH , Jakobsen M . Gut microbiota in human adults with type 2 diabetes differs from non-diabetic adults. PLoS One. 2010;5(2):e9085. doi: 10.1371/journal.pone.0009085. PMC2816710 20140211

[12] Limbana T , Khan F , Eskander N . Gut microbiome and depression: how microbes affect the way we think. Cureus. 2020;12(8):e9966. doi: 10.7759/cureus.9966. PMC7510518 32983670

[13] Liu X-Y , Li J , Zhang Y , Fan L , Xia Y , Wu Y , Chen J , Zhao X , Gao Q , Xu B , et al. Kidney microbiota dysbiosis contributes to the development of hypertension. Gut Microbes. 2022;14(1):2143220. doi: 10.1080/19490976.2022.2143220. PMC9662196 36369946

[14] Malesza IJ , Malesza M , Walkowiak J , Mussin N , Walkowiak D , Aringazina R , Bartkowiak-Wieczorek J , Mądry E . High-fat, Western-style diet, systemic inflammation, and gut microbiota: a narrative review. Cells. 2021;10(11):3164. doi: 10.3390/cells10113164. PMC8619527 34831387

[15] Tang WHW , Wang Z , Kennedy DJ , Wu Y , Buffa JA , Agatisa-Boyle B , Li XS , Levison BS , Hazen SL . Gut microbiota-dependent trimethylamine N-oxide (TMAO) pathway contributes to both development of renal insufficiency and mortality risk in chronic kidney disease. Circ Res. 2015;116(3):448–455. doi: 10.1161/CIRCRESAHA.116.305360. PMC4312512 25599331

[16] Lane G , Vatanparast H . Immigrant families’ experience of the Canadian food and nutrition environment. Appl Physiol Nutr Metab. 2023;48(6):445–454. doi: 10.1139/apnm-2022-0346. 36854137

[17] Cabieses B , Uphoff E , Pinart M , Antó JM , Wright J . A systematic review on the development of asthma and allergic diseases in relation to international immigration: the leading role of the environment confirmed. PLoS One. 2014;9(8):e105347. doi: 10.1371/journal.pone.0105347. PMC4139367 25141011

[18] Alidu L , Grunfeld EA . A systematic review of acculturation, obesity and health behaviours among migrants to high-income countries. Psychology & Health. 2018;33(6):724–745. doi: 10.1080/08870446.2017.1398327. 29172700

[19] Chatham RE , Mixer SJ . Cultural influences on childhood obesity in ethnic minorities: a qualitative systematic review. J Transcult Nurs. 2020;31(1):87–99. doi: 10.1177/1043659619869428. 31423926

[20] Agrawal M , Burisch J , Colombel JF , Shah S . Viewpoint: inflammatory bowel diseases among immigrants from low-to high-incidence countries: opportunities and considerations. J Crohn's Colitis. 2020;14(2):267–273. doi: 10.1093/ecco-jcc/jjz139. 31359034

[21] Flores GE , Caporaso JG , Henley JB , Rideout JR , Domogala D , Chase J , Leff JW , Vázquez-Baeza Y , Gonzalez A , Knight R , et al. Temporal variability is a personalized feature of the human microbiome. Genome Biol. 2014;15(12):531. doi: 10.1186/s13059-014-0531-y. PMC4252997 25517225

[22] Copeland JK , Chao G , Vanderhout S , Acton E , Wang PW , Benchimol EI , El-Sohemy A , Croitoru K , Gommerman JL , Guttman DS , GEMINI Research Team . The impact of migration on the gut metagenome of south asian Canadians. Gut Microbes. 2021;13(1):1902705. doi: 10.1080/19490976.2021.1902705. PMC8023248 33794735

[23] Vangay P , Johnson AJ , Ward TL , Al-Ghalith GA , Shields-Cutler RR , Hillmann BM , Lucas SK , Beura LK , Thompson EA , Till LM , et al. US immigration westernizes the human gut microbiome. Cell. 2018;175(4):962–972.e10. doi: 10.1016/j.cell.2018.10.029. PMC6498444 30388453

[24] Martínez I , Stegen JC , Maldonado-Gómez MX , Eren AM , Siba PM , Greenhill AR , Walter J . The gut microbiota of rural Papua New Guineans: composition, diversity patterns, and ecological processes. Cell Rep. 2015;11(4):527–538. doi: 10.1016/j.celrep.2015.03.049. 25892234

[25] Clemente JC , Pehrsson EC , Blaser MJ , Sandhu K , Gao Z , Wang B , Magris M , Hidalgo G , Contreras M , Noya-Alarcón Ó , et al. The microbiome of uncontacted amerindians. Sci Adv. 2015;1(3):e1500183. doi: 10.1126/sciadv.1500183. PMC4517851 26229982

[26] Sonnenburg ED , Sonnenburg JL . The ancestral and industrialized gut microbiota and implications for human health. Nat Rev Microbiol. 2019;17(6):383–390. doi: 10.1038/s41579-019-0191-8. 31089293

[27] Ifversen J . Who are the westerners? International Politics. 2008;45(3):236–253. doi: 10.1057/palgrave.ip.8800167.

[28] Kagan R . A tougher war for the US is one of legitimacy. 2004. https://archive.globalpolicy.org/empire/analysis/2004/0124legitimacy.htm

[29] Lieven A . The end of the West? Prospect. 2002. Prospect. https://www.prospectmagazine.co.uk/essays/59577/the-end-of-the-West.

[30] Aoki T . Aspects of globalization in contemporary Japan. In: Berger PL , Huntington SP , editors. Many Globalizations: Cultural diversity in the contemporary world. Oxford University Press; 2002; p. 68–88. doi: 10.1093/0195151461.003.0004.

[31] Bonnett A . The idea of the west: culture, politics and history. Palgrave MacMillan. 2004.

[32] Eid M , Karim KH . Public policy and the clash of ignorance. In: Karim IKH , Eid M , editors. Engaging the other: Public policy and Western-Muslim intersections. Palgrave Macmillan; 2014; p. 213–225. doi: 10.1057/9781137403698_11.

[33] Reyes-García V , Babigumira R , Pyhälä A , Wunder S , Zorondo-Rodríguez F , Angelsen A . Subjective wellbeing and income: empirical patterns in the rural developing world. J Happiness Stud. 2016;17(2):773–791. doi: 10.1007/s10902-014-9608-2. PMC5023045 27642259

[34] Khan MAH , Shahab M , Ali A , Ayaz N , Gul N , Khan A . Westernization vs. Indigenous traditions: a critical study of culture wars through the lens of cross-cultural communication and pragmatics. Annu Methodol Arch Res Rev. 2025;3(4):327–339.

[35] Lau H . The language of westernization in legal commentary. Am J Comp Law. 2013;61(3):507–538. doi: 10.5131/AJCL.2012.0024.

[36] Gollin D , Jedwab R , Vollrath D . Urbanization with and without industrialization. J Econ Growth. 2016;21(1):35–70. doi: 10.1007/s10887-015-9121-4.

[37] Winglee K , Howard AG , Sha W , Gharaibeh RZ , Liu J , Jin D , Fodor AA , Gordon-Larsen P . Recent urbanization in China is correlated with a westernized microbiome encoding increased virulence and antibiotic resistance genes. Microbiome. 2017;5:121. doi: 10.1186/s40168-017-0338-7. PMC5603068 28915922

[38] Yoneda M , Ohno H , Oki K . Westernization of lifestyle causes obesity-associated metabolic disorders in the Japanese population. J Obes Eat Disord. 2016;2(2):24. doi: 10.21767/2471-8203.100024.

[39] Chong-Neto HJ , D’amato G , Rosário Filho NA . Impact of the environment on the microbiome. J Pediatr. 2022;98:S32–S37. doi: 10.1016/j.jped.2021.10.001. PMC9510925 34742719

[40] Panthee B , Gyawali S , Panthee P , Techato K . Environmental and human microbiome for health. Life. 2022;12(3):456. doi: 10.3390/life12030456. PMC8949289 35330207

[41] Findley K , Williams DR , Grice EA , Bonham VL . Health disparities and the microbiome. TIM. 2016;24(11):847–850. doi: 10.1016/j.tim.2016.08.001. PMC5695575 27712950

[42] Ishaq SL , Parada FJ , Wolf PG , Bonilla CY , Carney MA , Benezra A , Wissel E , Friedman M , DeAngelis KM , Robinson JM , et al. Introducing the microbes and social equity working group: considering the microbial components of social, environmental, and health justice. mSystems. 2021;6(4):e0047121. doi: 10.1128/mSystems.00471-21. PMC8407420 34313460

[43] Kolářová K , Stöckelová T , Senft L . Disability and the (dysbiotic) gut: sensing, tasting and knowing with food. Sociology of Health & Illness. 2023;45(6):1242–1258. doi: 10.1111/1467-9566.13584. 36427010

[44] Benezra A . Race in the microbiome. Sci Technol Hum Values. 2020;45(5):877–902. doi: 10.1177/0162243920911998.

[45] Scaglioni M . Race by other names: critiquing population categories in microbiome research. mSystems. 2025;10(9):e00216-25. doi: 10.1128/msystems.00216-25. PMC12455957 40815145

[46] Núñez Casal A . Race and indigeneity in human microbiome science: microbiomisation and the historiality of otherness. Hist Philos Life Sci. 2024;46(2):17. doi: 10.1007/s40656-024-00614-w. PMC10987353 38565750

[47] Bader AC , Van Zuylen EM , Handsley-Davis M , Alegado RA , Benezra A , Pollet RM , Ehau-Taumaunu H , Weyrich LS , Anderson MZ . A relational framework for microbiome research with indigenous communities. Nat Microbiol. 2023;8(10):1768–1776. doi: 10.1038/s41564-023-01471-2. 37770743

[48] De Wolfe TJ , Arefin MR , Benezra A , Rebolleda Gómez M . Chasing ghosts: race, racism, and the future of microbiome research. mSystems. 2021;6(5):e00604–21. doi: 10.1128/mSystems.00604-21. PMC8510520 34636673

[49] Anukam KC , Reid G . African traditional fermented foods and probiotics. J Med Food. 2009;12(6):1177–1184. doi: 10.1089/jmf.2008.0163. 20041769

[50] O'Keefe SJD . Nutrition and colonic health: the critical role of the microbiota. Curr Opin Gastroenterol. 2008;24(1):51–58. doi: 10.1097/MOG.0b013e3282f323f3. 18043233

[51] Latella G , Fiocchi C , Caprili R . News from the “5th international meeting on inflammatory bowel diseases” CAPRI 2010. Journal of Crohn's and Colitis. 2010;4(6):690–702. doi: 10.1016/j.crohns.2010.08.002. 21122584

[52] D’Aloisio LD , Ballal M , Ghosh S , Haskey N , Abulizi N , Karimianghadim R , Liu C , Sruthi P , Nagarajan L , Vasudevan S , et al. The adoption of a westernized gut microbiome in Indian immigrants and indo-Canadians is associated with dietary acculturation. NPJ Biofilms Microbiomes. 2025;11:151. doi: 10.1038/s41522-025-00778-8. PMC12317999 40753175

[53] Peters BA , Yi SS , Beasley JM , Cobbs EN , Choi HS , Beggs DB , Hayes RB , Ahn J . US nativity and dietary acculturation impact the gut microbiome in a diverse US population. ISME J. 2020;14(7):1639–1650. doi: 10.1038/s41396-020-0630-6. PMC7305110 32210364

[54] Guo J , Zhang X , Saiganesh A , Peacock C , Filipovska-Naumovska E , Chen S , Khoo S-K , Dykes G , Hales B , Le Souëf P , et al. Linking the westernised oropharyngeal microbiome to the innate and adaptive immune response in Chinese immigrants. World Allergy Organ J. 2020;13(8):100164. doi: 10.1016/j.waojou.2020.100164. PMC7491349 32944027

[55] Kaplan RC , Wang Z , Usyk M , Sotres-Alvarez D , Daviglus ML , Schneiderman N , Talavera GA , Gellman MD , Thyagarajan B , Moon J-Y , et al. Author correction: gut microbiome composition in the hispanic community health Study/Study of latinos is shaped by geographic relocation, environmental factors, and obesity. Genome Biol. 2020;21(1):50. doi: 10.1186/s13059-019-1831-z. PMC7043042 32098632

[56] Sonnenburg JL , Sonnenburg ED . Vulnerability of the industrialized microbiota. Sci. 2019;366(6464):eaaw9255. doi: 10.1126/science.aaw9255. 31649168

[57] van der Vossen EWJ , Davids M , Bresser LRF , Galenkamp H , van den Born BH , Zwinderman AH , Levin E , Nieuwdorp M , de Goffau MC . Gut microbiome transitions across generations in different ethnicities in an urban setting-the HELIUS study. Microbiome. 2023;11(1):99. doi: 10.1186/s40168-023-01488-z. PMC10165778 37158898

[58] D’Aloisio LD , Shetty V , Ballal M , Gibson DL . Following the Indian immigrant: adoption of westernization results in a Western gut microbiome and an increased risk of inflammatory bowel diseases. FEMS Microbiol Ecol. 2022;98(12):fiac133. doi: 10.1093/femsec/fiac133. 36370451

[59] David LA , Maurice CF , Carmody RN , Gootenberg DB , Button JE , Wolfe BE , Ling AV , Devlin AS , Varma Y , Fischbach MA , et al. Diet rapidly and reproducibly alters the human gut microbiome. Natur. 2014a;505(7484):559–563. doi: 10.1038/nature12820. PMC3957428 24336217

[60] David LA , Materna AC , Friedman J , Campos-Baptista MI , Blackburn MC , Perrotta A , Erdman SE , Alm EJ . Host lifestyle affects human microbiota on daily timescales. Genome Biol. 2014b;15(7):R89. doi: 10.1186/gb-2014-15-7-r89. PMC4405912 25146375

[61] Azzam A . Is the world converging to a “Western diet”? Public Health Nutr. 2021;24(2):309–317. doi: 10.1017/S136898002000350X. PMC10195558 33077024

[62] O’Keefe SJD , Li JV , Lahti L , Ou J , Carbonero F , Mohammed K , Posma JM , Kinross J , Wahl E , Ruder E , et al. Fat, fibre and cancer risk in African Americans and rural africans. Nat Commun. 2015;6(1):6342. doi: 10.1038/ncomms7342. PMC4415091 25919227

[63] Wang Y , Chen G-C , Wang Z , Luo K , Zhang Y , Li Y , McClain AC , Jankowska MM , Perreira KM , Mattei J , et al. Dietary acculturation is associated with altered gut microbiome, circulating metabolites, and cardiovascular disease risk in US hispanics and latinos: results from HCHS/SOL. Circulation. 2024;150(3):215–229. doi: 10.1161/CIRCULATIONAHA.124.069824. PMC11460527 39008559

[64] Boolchandani M , Blake KS , Tilley DH , Cabada MM , Schwartz DJ , Patel S , Morales ML , Meza R , Soto G , Isidean SD , et al. Impact of international travel and diarrhea on gut microbiome and resistome dynamics. Nat Commun. 2022;13(1):7485. doi: 10.1038/s41467-022-34862-w. PMC9722912 36470885

[65] Dey N , Wagner VE , Blanton LV , Cheng J , Fontana L , Haque R , Ahmed T , Gordon JI . Regulators of gut motility revealed by a gnotobiotic model of diet-microbiome interactions related to travel. Cell. 2015;163(1):95–107. doi: 10.1016/j.cell.2015.08.059. PMC4583712 26406373

[66] Howard AG , Attard SM , Herring AH , Wang H , Du S , Gordon-Larsen P . Socioeconomic gradients in the westernization of diet in China over 20 years. SSM - Population Health. 2021;16:100943. doi: 10.1016/j.ssmph.2021.100943. PMC8526760 34703875

[67] He Y , Yang X , Xia J , Zhao L , Yang Y . Consumption of meat and dairy products in China: a review. Proc Nutr Soc. 2016;75(3):385–391. doi: 10.1017/S0029665116000641. 27334652

[68] Ouyang Y , Tan T , Song X , Huang F , Zhang B , Ding G , Wang H . Dietary protein intake dynamics in elderly Chinese from 1991 to 2018. Nutrients. 2021;13(11):3806. doi: 10.3390/nu13113806. PMC8622550 34836062

[69] Shin J-H , Jung S , Kim S-A , Kang M-S , Kim M-S , Joung H , Hwang G-S , Shin D-M . Differential effects of typical Korean versus American-style diets on gut microbial composition and metabolic profile in healthy overweight Koreans: a randomized crossover trial. Nutrients. 2019;11(10):2450. doi: 10.3390/nu11102450. PMC6835328 31615057

[70] Nadkarni SC . Ethnic product usage in a multicultural Canada: Influences of identity, acculturation and access. Atrium. 2026. [Doctoral dissertation, University of Guelph]. https://atrium.lib.uoguelph.ca/handle/10214/151.

[71] Lee SD , Kellow NJ , Choi TST , Huggins CE . Assessment of Dietary Acculturation in East Asian Populations: A Scoping Review. Adv Nutr. 2021;12(3):865–886. doi: 10.1093/advances/nmaa127. PMC8166541 33119743

[72] BaHammam AS , Pirzada A . Timing matters: the interplay between early mealtime, circadian rhythms, gene expression, circadian hormones, and metabolism—a narrative review. Clocks & Sleep. 2023;5(3):507–535. doi: 10.3390/clockssleep5030034. PMC10528427 37754352

[73] Hauptmann AL , Paulová P , Castro-Mejía JL , Hansen LH , Sicheritz-Pontén T , Mulvad G , Nielsen DS . The microbial composition of dried fish prepared according to greenlandic inuit traditions and industrial counterparts. Food Microbiol. 2020;85:103305. doi: 10.1016/j.fm.2019.103305. 31500717

[74] Barra NG , Fang H , Bhatwa A , Schmidt AM , Syed SA , Steinberg GR , Morrison KM , Surette MG , Wade MG , Holloway AC , et al. Food supply toxicants and additives alter the gut microbiota and risk of metabolic disease. Am J Physiol Endocrinol Metab. 2025;328(3):E337–E353. doi: 10.1152/ajpendo.00364.2024. 39871724

[75] Marshall Q , Fanzo J , Barrett CB , Jones AD , Herforth A , McLaren R . Building a global food systems typology: a new tool for reducing complexity in food systems analysis. Front Sustain Food Syst. 2021;5:746512. doi: 10.3389/fsufs.2021.746512.

[76] Baker P , Machado P , Santos T , Sievert K , Backholer K , Hadjikakou M , Russell C , Huse O , Bell C , Scrinis G , et al. Ultra-processed foods and the nutrition transition: global, regional and national trends, food systems transformations and political economy drivers. Obesity Reviews. 2020;21(12):e13126. doi: 10.1111/obr.13126. 32761763

[77] Kim S , Zhang W , Pak V , Aqua JK , Hertzberg VS , Spahr CM , Slavich GM , Bai J . How stress, discrimination, acculturation and the gut microbiome affect depression, anxiety and sleep among Chinese and Korean immigrants in the USA: a cross-sectional pilot study protocol. BMJ Open. 2021;11(7):e047281. doi: 10.1136/bmjopen-2020-047281. PMC8296781 34290066

[78] Merra G , Gualtieri P , La Placa G , Frank G , Della Morte D , De Lorenzo A , Di Renzo L . The relationship between exposome and microbiome. Microorganisms. 2024;12(7):1386. doi: 10.3390/microorganisms12071386. PMC11278511 39065154

[79] Amit K , Riss I . The subjective well-being of immigrants: pre- and post-migration. Soc Indic Res. 2014;119(1):247–264. doi: 10.1007/s11205-013-0492-7.

[80] Bekteshi V , Kang S-W . Contextualizing acculturative stress among latino immigrants in the United States: a systematic review. Ethnicity & Health. 2020;25(6):897–914. doi: 10.1080/13557858.2018.1469733. 29792072

[81] Lasser KE , Himmelstein DU , Woolhandler S . Access to care, health status, and health disparities in the United States and Canada: results of a cross-national population-based survey. Am J Public Health. 2006;96(7):1300–1307. doi: 10.2105/AJPH.2004.059402. PMC1483879 16735628

[82] Berggreen-Clausen A , Pha SH , Alvesson HM , Andersson A , Daivadanam M . Food environment interactions after migration: a scoping review on low- and middle-income country immigrants in high-income countries. Public Health Nutr. 2022;25(1):136–158. doi: 10.1017/S1368980021003943. PMC8825972 34509180

[83] Jones-Antwi RE , Judd SE , Cunningham SA . Food choices during resettlement among immigrants in the US. BMC Public Health. 2025;25(1):2184. doi: 10.1186/s12889-025-22986-9. PMC12220798 40604660

[84] Bennett G , Bardon LA , Gibney ER . A comparison of dietary patterns and factors influencing food choice among ethnic groups living in one locality: a systematic review. Nutrients. 2022;14(5):941. doi: 10.3390/nu14050941. PMC8912306 35267916

[85] Cooksey Stowers K , Jiang Q , Atoloye A , Lucan S , Gans K . Racial differences in perceived food swamp and food desert exposure and disparities in self-reported dietary habits. Int J Environ Res Public Health. 2020;17(19):7143. doi: 10.3390/ijerph17197143. PMC7579470 33003573

[86] Diener C , Holscher HD , Filek K , Corbin KD , Moissl-Eichinger C , Gibbons SM . Metagenomic estimation of dietary intake from human stool. Nature Metabolism. 2025;7(3):617–630. doi: 10.1038/s42255-025-01220-1. PMC11949708 39966520

[87] Farmer N , Maki KA , Barb JJ , Jones KK , Yang L , Baumer Y , Powell-Wiley TM , Wallen GR . Geographic social vulnerability is associated with the alpha diversity of the human microbiome. mSystems. 2023;8(5):e0130822. doi: 10.1128/msystems.01308-22. PMC10654076 37642431

[88] Fouladi F , Bailey MJ , Patterson WB , Sioda M , Blakley IC , Fodor AA , Jones RB , Chen Z , Kim JS , Lurmann F , et al. Air pollution exposure is associated with the gut microbiome as revealed by shotgun metagenomic sequencing. Environ Int. 2020;138:105604. doi: 10.1016/j.envint.2020.105604. PMC7181344 32135388

[89] Nielsen CC , Gascon M , Osornio-Vargas AR , Shier C , Guttman DS , Becker AB , Azad MB , Sears MR , Lefebvre DL , Moraes TJ , et al. Natural environments in the urban context and gut microbiota in infants. Environ Int. 2020;142:105881. doi: 10.1016/j.envint.2020.105881. 32610248

[90] Benezra A . Gut anthro. University of Minnesota Press; 2023.

[91] Roberts EFS , Goodrich JM , Jansen EC , Needham BL , Sánchez BN , Téllez Rojo MM . Bioethnography. In: Pentecost IM , Keaney J , Moll T , Penkler M , editors. The handbook of DOHaD and society. Cambridge University Press; 2024a; p. 174–184. doi: 10.1017/9781009201704.017.

[92] Pasolli E , Schiffer L , Manghi P , Renson A , Obenchain V , Truong DT , Beghini F , Malik F , Ramos M , Dowd JB , et al. Accessible, curated metagenomic data through ExperimentHub. Nature Methods. 2017;14(11):1023–1024. doi: 10.1038/nmeth.4468. PMC5862039 29088129

[93] Ramos Sarmiento K , Carr A , Diener C , Locey KJ , Gibbons SM . Island biogeography theory provides a plausible explanation for why larger vertebrates and taller humans have more diverse gut microbiomes. ISME J. 2024;18(1):wrae114. doi: 10.1093/ismejo/wrae114. PMC11253425 38904949

[94] Yatsunenko T , Rey FE , Manary MJ , Trehan I , Dominguez-Bello MG , Contreras M , Magris M , Hidalgo G , Baldassano RN , Anokhin AP , et al. Human gut microbiome viewed across age and geography. Natur. 2012;486(7402):222–227. doi: 10.1038/nature11053. PMC3376388 22699611

[95] Johnson-Martínez JP , Diener C , Levine AE , Wilmanski T , Suskind DL , Ralevski A , Hadlock J , Magis AT , Hood L , Rappaport N , et al. Aberrant bowel movement frequencies coincide with increased microbe-derived blood metabolites associated with reduced organ function. Cell Reports Medicine. 2024;5(7):101646. doi: 10.1016/j.xcrm.2024.101646. PMC11293344 39019013

[96] Asnicar F , Leeming ER , Dimidi E , Mazidi M , Franks PW , Al Khatib H , Valdes AM , Davies R , Bakker E , Francis L , et al. Blue poo: impact of gut transit time on the gut microbiome using a novel marker. Gut. 2021;70(9):1665–1674. doi: 10.1136/gutjnl-2020-323877. PMC8349893 33722860

[97] Sun W , Zhang Y , Guo R , Sha S , Chen C , Ullah H , Zhang Y , Ma J , You W , Meng J , et al. A population-scale analysis of 36 gut microbiome studies reveals universal species signatures for common diseases. NPJ Biofilms Microbiomes. 2024;10:96. doi: 10.1038/s41522-024-00567-9. PMC11442664 39349486

[98] Wasney M , Briscoe L , Wolff R , Ghezzi H , Tropini C , Garud N . Uniform bacterial genetic diversity along the gut. Nat Commun. 2026;17(1):4100. doi: 10.1038/s41467-026-70705-8. PMC13144417 41844646

